# Quantitative analysis of intrarenal nerve‐associated profiles in the human foetal kidney between 14 and 30 gestational weeks: An S100 immunohistochemical study

**DOI:** 10.1111/joa.70260

**Published:** 2026-10-14

**Authors:** Ivan Petrović, Ana Balenović, Milana Bosanac, Srdjan Stanković, Isidora Milosavljević, Milena Šunjević, Sandra Trivunić Dajko, Bojana Andrejić‐Višnjić

**Affiliations:** ^1^ High‐Field MR Center, Department of Biomedical Imaging and Image‐Guided Therapy Medical University of Vienna Vienna Austria; ^2^ Department of Neurology Medical University of Vienna Vienna Austria; ^3^ Faculty of Medicine University of Novi Sad Novi Sad Serbia; ^4^ Institute of Cardiovascular Diseases of Vojvodina Sremska Kamenica Serbia; ^5^ Faculty of Medicine Semmelweis University Budapest Hungary

**Keywords:** foetal kidney, intrarenal innervation, nephrogenesis, S100 immunohistochemistry

## Abstract

Quantitative data on the spatial development of intrarenal S100‐positive nerve‐associated profiles in human foetal kidneys remain limited, despite the established importance of renal nerves. Building on the previously published semiquantitative observations, this study aimed to generate a quantitative, compartment‐resolved map of S100‐positive nerve‐associated profiles in human foetal kidneys. Formalin‐fixed, paraffin‐embedded human foetal kidney specimens ranging from 14 to 30 gestational weeks were analysed using S100 immunohistochemistry. Based on gestational age, specimens were divided into three groups: ≤18 gestational weeks (G1, n=6), 19–24 gestational weeks (G2, n=6), and ≥ 25 gestational weeks (G3, n=6). Cortical maturation was assessed by measuring nephrogenic zone and maturation zone thickness and calculating the cortical maturation index (CMI). Morphometric analysis of cortical S100‐positive nerve‐associated profiles was performed using digital image analysis. Distribution patterns of S100‐positive profiles were assessed qualitatively. Group‐wise analysis demonstrated significantly lower cortical S100‐positive area in G1 compared with G2 and G3 (median: 985 μm^2^ vs. 1733 μm^2^ and 2400 μm^2^, respectively; *p* < 0.001). Cortical S100‐positive area increased significantly with gestational age and foetal weight and showed an inverse correlation with CMI (*ρ* = −0.410, *p* < 0.001), indicating parallel progression of S100‐positive area and cortical maturation. In multivariate linear regression analyses, cortical S100‐positive area remained independently associated with gestational age (*β* = 125.0, *p* = 0.006) and foetal weight (*β* = 1.50, *p* = 0.006), explaining 57.6% of the variance (adjusted *R* ^2^ = 0.485). Perivascular localisation was the most frequently observed distribution pattern, while juxtaglomerular and intraglomerular patterns were also identified, including in the earliest gestational‐age group. This study provides the first quantitative, compartment‐resolved analysis of intrarenal nerve‐associated profiles in the human foetal kidney. Cortical S100‐positive area increases with gestational age in parallel with cortical maturation and shows a predominantly perivascular distribution, consistent with a neurovascular co‐development model and establishing a morphological framework for future functional studies of renal innervation.

## Graphical abstract

S100 immunohistochemistry and digital morphometry were used to characterise nerve‐associated profiles in human foetal kidneys from 14 to 30 gestational weeks. Cortical S100‐positive area increased with gestational age and was positively associated with foetal weight and maturation zone thickness. Predominantly perivascular localisation highlights the spatial relationship between S100‐positive nerve‐associated profiles and the developing renal vasculature.

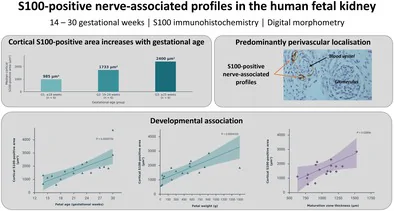

## 1 INTRODUCTION

As the central organs of the urinary system, the kidneys play a pivotal role in cardiovascular homeostasis via autonomic regulation of renal function (Jackson, 2004), (Johns, 2013). Renal innervation is predominantly sympathetic, comprising both efferent postganglionic and afferent fibres (Robillard & Nakamura, 1988), and is mainly adrenergic (Johns et al., 2011), with a smaller acetylcholinesterase‐positive component (Barajas & Wang, 1975). Contrasting this, parasympathetic innervation of the kidney parenchyma appears limited and has been attributed to vagal sources (Tiniakos et al., 2004). Functionally, renal nerves regulate renin secretion from juxtaglomerular granular cells, tubular salt handling, and renal vascular tone (DiBona, 2000; DiBona, 2003). Experimental evidence also supports a role for renal nerves in kidney development and maturation, including effects on foetal renal haemodynamics and nephrogenic signalling pathways (Robillard et al., 1993; Slotkin et al., 1987; Slotkin et al., 1988), transition from foetal to newborn period (Page et al., 1992; Smith et al., 1991; Smith et al., 1992), and influence on embryogenesis and tubular differentiation through the production of neurotrophic growth factors (Robillard et al., 1993; Sariola et al., 1991).

Historically, the majority of research, including foetal kidney material, was done on animal models. To date, the first semiquantitative analysis of the developing kidney innervation in human foetuses was done by Tiniakos et al. (Tiniakos et al., 2004), and by this, a monumental contribution was made to the growing body of knowledge on this topic. Their study included foetal kidney material from the second and third trimester, with the youngest specimen being 20 gestational weeks (GW) old. Some of the main findings were that the majority of renal nerves have perivascular distribution, and the development of renal efferent sympathetic innervation is coupled with the growth of the vasculature, which shows the centrifugal fashion of development, reaching the adult level of innervation at 28GW (Tiniakos et al., 2004), (Gattone et al., 1990). As their work was semiquantitative, the results, although valuable, could be improved by exact quantification. One marker used to visualise nerve‐associated structures is S100, which labels Schwann cells and other glial‐lineage elements and is therefore commonly used to highlight peripheral nerve profiles in tissue sections (S100 [Internet], 2026; Steiner et al., 2007). Prior immunohistochemical work has reported S100B negativity in several non‐neural renal/interstitial cell populations (e.g., endothelial and inflammatory cells), supporting its utility for identifying nerve‐associated profiles in this context (Dourou et al., 2006).

Despite the recognised importance of renal innervation for kidney function and maturation, quantitative data on the spatial development of intrarenal S100‐positive nerve‐associated profiles in human foetal kidneys remain scarce. Building on the seminal semiquantitative observations by Tiniakos et al., we sought to provide a quantitative, compartment‐resolved map of developing intrarenal S100‐positive nerve‐associated profiles in human foetuses.

## 2 MATERIALS AND METHODS

### 2.1 Sample collection

The research was conducted at the Department of Histology and Embryology of the Faculty of Medicine, University of Novi Sad, Serbia, and it was approved by the Ethics Committee of the University Clinical Center of Vojvodina, Novi Sad, Serbia (No. 00–1160). Formalin‐fixed paraffin‐embedded (FFPE) blocks of autopsied foetal kidney tissue, collected during a 2‐year period, were obtained from the archive of the Center for Pathology and Histology of the aforementioned University Clinical Center. The specimens had to fulfil the following inclusion criteria: (1) congenital malformations of the foetus were absent both on the ultrasound performed before the abortion and during the autopsy, (2) no clinical suspicion of chromosomal abnormality, (3) gross absence of maceration and autolysis of the kidney tissue in histological slides, (4) presence of the capsule on the kidney surface to ensure cortical representation and (5) successful immunohistological staining with the S100 antibody. A total of 18 foetal kidney specimens met the predefined inclusion criteria and were included in the study.

Data on the anthropometric parameters of foetuses, whose kidney tissue material was included in the analysis, were retrieved from the archive of the Center for Pathology and Histology at the Clinical Center of Vojvodina. The anthropometric parameters obtained from the autopsy records were foetal weight expressed in grams (g) and gestational age in GWs. Based on gestational age, the 18 specimens were divided into three balanced strata: G1 comprised specimens from foetuses aged 14–18 GWs (*n* = 6), G2 comprised those aged 19–24 GWs (*n* = 6), and G3 comprised those aged 25–30 GWs (*n* = 6). The selected intervals are closely aligned with gestational stratification previously applied in histological studies of the developing human kidney, in which changes in corticomedullary differentiation and tubular and medullary maturation were described across comparable gestational intervals (Pokarna et al., 2019). The strata also permitted group‐based comparisons while maintaining equal group sizes. Because renal development is continuous, the cut‐offs were not regarded as boundaries between discrete biological stages; gestational age was additionally analysed as a continuous numerical variable in the correlation and regression analyses.

### 2.2 Histological and immunohistochemical staining

After the selection of samples, two 5 μm thick sections were obtained from each block. One tissue section was stained with haematoxylin and eosin (HE) while the other underwent immunohistochemistry (IHC). For the IHC, the histological slides with tissue sections were stained following the manufacturer's instructions for the manual protocol with the EnVision™ FLEX System. The antibody used for the study was S100 (Abcam, AB52642, 1:1000). Both positive and negative external controls were performed for quality control. The immunohistochemical staining aimed to visualise S100‐positive nerve‐associated profiles (fibres/bundles) in the parenchyma of the foetal kidney.

### 2.3 Morphometric analysis of the renal cortex

Morphometric analysis was performed using ImageJ (NIH, Bethesda, MD, USA) (Schneider et al., 2012), a public‐domain software for scientific image analysis. Images were acquired using a Leica DM LB light microscope equipped with a Leica DC100 digital camera (Leica Biosystems, IL, USA). Prior to any measurements, all images were spatially calibrated in ImageJ (Analyse → Set Scale) using the microscope/camera calibration. For each kidney, the renal cortex on H&E‐stained sections was sampled using five non‐overlapping fields of view acquired at 100× magnification. Fields were selected to span the full cortical thickness so that each image included the cortex from the renal capsule to the corticomedullary junction. These images were used to measure nephrogenic zone thickness (NZ) and maturation zone thickness (MZ). NZ thickness was measured as the linear distance from the inner surface of the renal capsule to the proximal pole (distal extent) of the outermost S‐shaped bodies, using the ‘Straight line’ tool in ImageJ. MZ thickness was measured from the NZ boundary (defined at the level of S‐shaped bodies) to the corticomedullary junction, also using the ‘Straight line’ tool (Figure 1a) (Andrejić et al., 2024; Ryan et al., 2018). All measurements were recorded in micrometers (μm). The cortical maturation index (CMI) was calculated as the ratio NZ/MZ and reported as an absolute value. Higher CMI values indicate a relatively wider NZ compared with the MZ and therefore a lower degree of cortical maturation; accordingly, CMI decreases with increasing gestational age (Andrejić et al., 2024).

**FIGURE 1:**
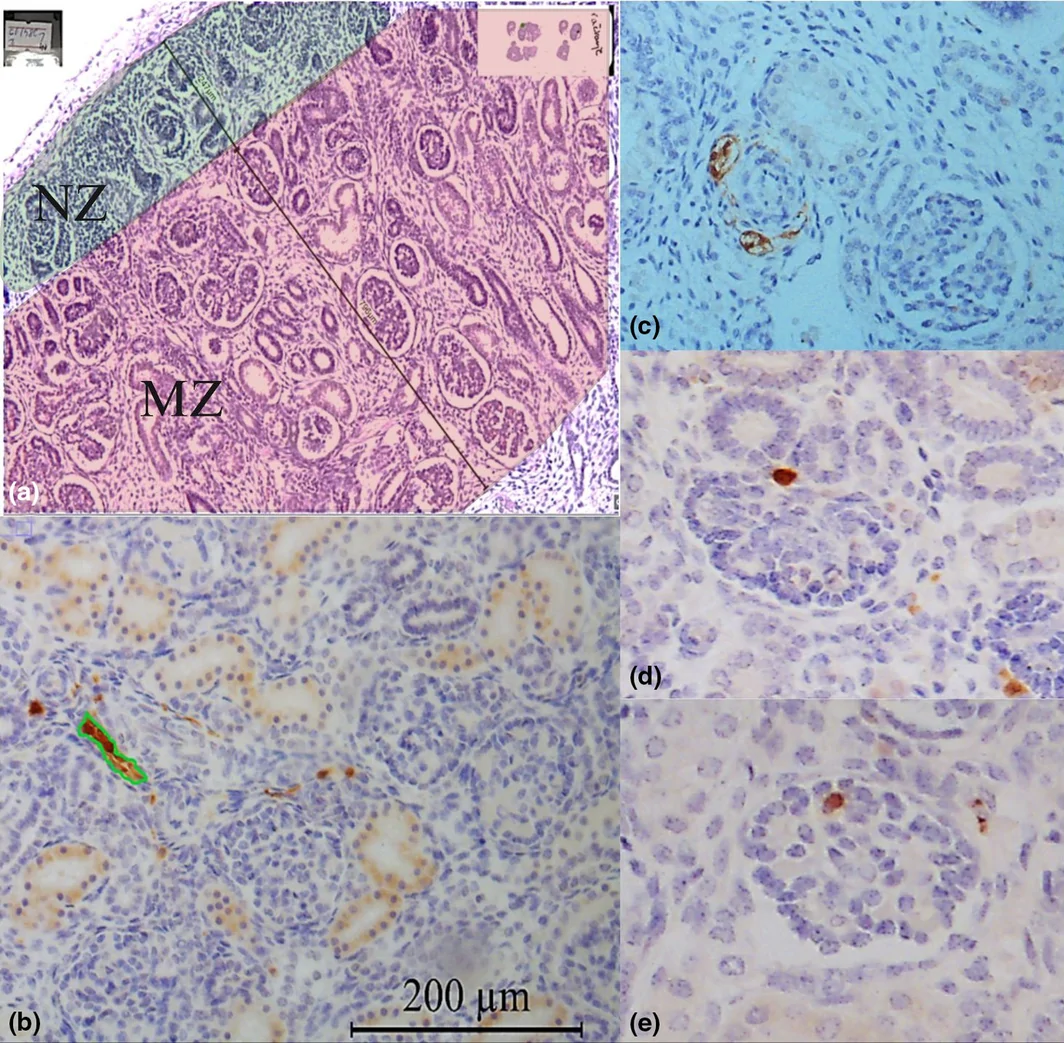
Histological analysis. The thickness of the nephrogenic zone (NZ; green overlay) and maturation zone (MZ; red overlay) was evaluated on H&E‐stained sections (a). S100‐immunostained sections were used to quantify the cortical S100‐positive area (b) and to qualitatively assess the cortical distribution patterns of S100‐positive profiles, including perivascular (c), juxtaglomerular (d) and intraglomerular (e) localisation.

### 2.4 Analysis of the S100 ^+^ fibres and cells

Immunohistochemically stained sections were examined to assess S100‐positive nerve‐associated profiles in the renal cortex. Using the same microscope/camera setup and imaging approach as described above, five non‐overlapping cortical fields per kidney were acquired at 100× magnification, with each field spanning from the renal capsule to the corticomedullary junction. All images were analysed on calibrated ImageJ measurements (Analyse → Set Scale), and measurements were recorded in square micrometers (μm^2^).

#### 2.4.1 Measurement of the S100 ^+^ area of the cortex

For each cortical field, the S100‐positive signal within the cortex was quantified as an area. In ImageJ, S100‐positive nerve‐associated profiles (fibres/bundles) and S100‐positive cells within the cortical region of interest were manually outlined using the ‘Freehand selection’ tool by tracing a closed contour along the visible immunopositive reaction product (3,3′‐diaminobenzidine, DAB), and the enclosed area was recorded (Figure 1b). The total cortical S100‐positive area per kidney (Area‐S100+) was calculated as the mean of the measured areas across the five fields and expressed in μm^2^.

#### 2.4.2 Analysis of the distribution patterns of the S100 ^+^ cells and fibres in the cortex

The distribution of S100‐positive nerve‐associated profiles in the renal cortex was assessed in a qualitative manner. Using the same five non‐overlapping cortical fields acquired per specimen (capsule to corticomedullary junction), S100‐positive profiles were evaluated for their predominant localisation pattern. Each profile was classified into one of the following categories: (i) perivascular (in direct contact with or immediately surrounding a blood vessel wall), (ii) juxtaglomerular (in the vicinity of the glomerular vascular pole/juxtaglomerular apparatus region), or (iii) intraglomerular (within the glomerular tuft) (Figure 1c–e, respectively). For each specimen, the relative prominence of distribution patterns was assessed qualitatively across the analysed cortical fields, allowing identification of the most frequently observed localisation pattern without formal quantification.

### 2.5 Statistical analysis

Statistical analyses were performed using the publicly available software *Jamovi* (version 2.4.1). Normality of continuous variables was assessed using the Shapiro–Wilk test. Because assumptions of normality were not met, differences among gestational‐age groups were evaluated using the Kruskal–Wallis test, followed by Dwass–Steel–Critchlow–Fligner pairwise comparisons for post hoc testing. A two‐tailed *p*‐value < 0.05 was considered statistically significant. Results are presented as median and interquartile range (IQR). Univariate simple linear regressions (*α* = 0.05) and Spearman rank correlations, followed by multiple linear regression with significant univariate predictors, were performed to evaluate associations of S100 positivity area in the cortex with gestational age, foetal weight, NZ and MZ proportions and CMI.

## 3 RESULTS

### 3.1 Sample characteristics and cortical maturation

The study included 18 foetal kidney FFPE blocks ranging from 14 to 30 GWs (Table 1). NZ thickness differed significantly among the groups (Kruskal–Wallis, *p* < 0.001). Post hoc Dwass–Steel–Critchlow–Fligner analysis demonstrated that NZ thickness was significantly lower in G3 compared with both G1 and G2. MZ thickness also showed significant differences among groups (Kruskal–Wallis, *p* < 0.001), with post hoc analysis revealing significantly lower MZ thickness in G1 compared with G2 and G3. The CMI differed significantly among groups (Kruskal–Wallis, *p* < 0.001) and exhibited a progressive decline with advancing gestational age. Post hoc testing confirmed that CMI was significantly lower in G3 compared with G1 and G2 (Table 1).

**TABLE 1:** Overview of the anthropometric parameters, cortical maturation parameters and index.

Group	Gestational age (GW)	Mass (g)	NZ (μm)	MZ (μm)	CMI
G1 (*n* = 6)	15.7 ± 1.63	50 (IQR 40–75)	194 (IQR 158–254)	789 (IQR 620–996)*	0.25 (IQR 0.17–0.33)
G2 (*n* = 6)	21.8 ± 1.83	470 (IQR 330–550)	200 (IQR 156–234)	1127 (IQR 940–1234)	0.19 (IQR 0.16–0.23)
G3 (*n* = 6)	28.2 ± 1.94	880 (IQR 765–1280)	143 (IQR 121–171)*	1107 (IQR 1023–1284)	0.13 (IQR 0.09–0.16)*

*Note*: Data are median (IQR) and mean values ± standard deviation.

Abbreviations: CMI, cortical maturation index (NZ/MZ); GW, gestational weeks; IQR, interquartile range; MZ, maturation zone thickness; NZ, nephrogenic zone thickness.

* *p* < 0.05 for indicated pairwise comparisons (Dwass–Steel–Critchlow–Fligner).

### 3.2 Cortical S100‐positive area

S100‐positive area in the cortex increased with gestational age. Group comparisons revealed significant differences among gestational age groups (*p* < 0.001), with G1 exhibiting lower values than G2 and G3 on post hoc testing (Table 2). Spearman correlation analyses demonstrated strong positive associations with gestational age (*ρ* = 0.849, *p* < 0.001) and foetal weight (*ρ* = 0.833, *p* < 0.001), as well as a moderate negative association with the corticomedullary index (CMI; *ρ* = −0.410, *p* < 0.001).

**TABLE 2:** Cortical S100‐positive area by gestational‐age group.

Group	Cortical S100‐positive area (μm^2^)
G1	985 (IQR 532–1258)*
G2	1733 (IQR 1323–2439)
G3	2400 (IQR 1347–4517)

Abbreviation: IQR, interquartile range.

* *p* < 0.01 for indicated pairwise comparisons (Dwass–Steel–Critchlow–Fligner).

Univariate linear regression analyses identified gestational age (*F*(1,16) = 19.128, *p* < 0.001), foetal weight (*F*(1,16) = 19.663, *p* < 0.001), and MZ thickness (*F*(1,16) = 5.058, *p* = 0.039) as significant predictors of cortical S100‐positive area, whereas NZ thickness (*F*(1,16) = 1.909, *p* = 0.186) and CMI (*F*(1,16) = 3.766, *p* = 0.070) were not (Figure 2).

**FIGURE 2:**
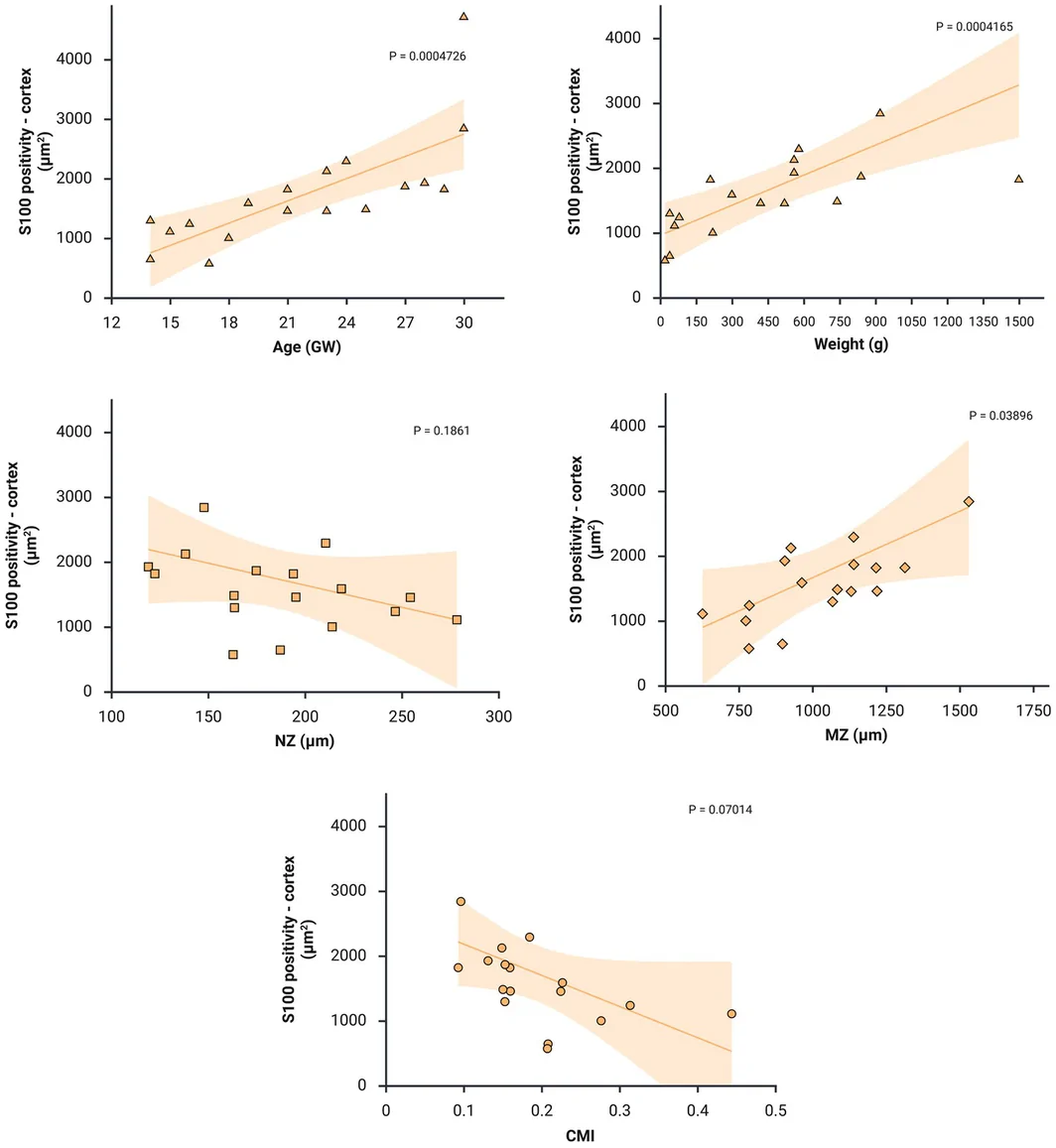
Analysis of S100‐immunostained kidney tissue. Univariate linear regression analyses demonstrate associations between cortical S100‐positive area and gestational age, foetal weight, nephrogenic zone thickness, maturation zone thickness and cortical maturation index. Solid lines represent linear regression fits, with shaded areas indicating 95% confidence intervals. CMI, cortical maturation index; GW, gestational weeks; MZ, maturation zone; NZ, nephrogenic zone.

In multiple linear regression analyses, cortical S100‐positive area remained associated with age (*β* = 125.0, 95% CI: 40.8–209.2, *p* = 0.006) and foetal weight (*β* = 1.50, 95% CI: 0.51–2.49, *p* = 0.006) when adjusted for MZ thickness. However, in the fully adjusted model including all three variables, no predictor remained independently significant (all *p* > 0.30), with the model explaining 57.6% of the variance in cortical S100‐positive area (adjusted *R* ^2^ = 0.485).

#### 3.2.1 Distribution of S100‐positive profiles in the cortex

Perivascular distribution was consistently observed, while juxtaglomerular and intraglomerular patterns were also identified, including in the earliest gestational‐age group.

## 4 DISCUSSION

To the best of our knowledge, this is the first quantitative, compartment‐resolved analysis of intrarenal S100‐positive nerve‐associated profiles development in the human foetal kidney across mid‐ to late gestation. Using S100 immunohistochemistry, we provide a systematic assessment of the spatial expansion and maturation of nerve‐associated profiles in the renal cortex between 14 and 30 GWs. Our results demonstrate a progressive increase in cortical S100‐positive area with advancing gestational age and foetal growth, accompanied by a parallel decline in NZ thickness and CMI. These findings support a close temporal coupling between cortical maturation and intrarenal neural development in the human foetal kidney. In addition, the predominance of perivascular nerve‐associated profiles, together with the early appearance of juxtaglomerular and intraglomerular patterns, underscores the intimate relationship between neural, vascular and nephron maturation during human renal development.

### 4.1 Expansion of cortical S100‐positive nerve‐associated profiles parallels cortical maturation

Quantitative assessment demonstrated that the expansion of intrarenal S100‐positive nerve‐associated profiles in the renal cortex is closely associated with advancing gestational age, which emerged as the strongest predictor of cortical S100‐positive area, reflecting a progressive increase in nerve‐associated profiles across mid‐ to late gestation. This age‐related increase occurs in parallel with histomorphological cortical maturation, including regression of the NZ and expansion of the MZ, as summarised by the CMI (Andrejić et al., 2024). Our findings also align with prior human immunohistochemical observations that cortical intrarenal nerves become readily detectable from around 20 GWs and increase towards term (Tiniakos et al., 2004), supporting the concept that maturation of the cortical microenvironment is accompanied by a progressive elaboration of the neural network. From a structural perspective, this coupling may reflect the dependence of intrarenal nerve growth on the maturation of the cortical microenvironment, including vascular remodelling and zonal differentiation. As cortical compartments mature and the NZ regresses, the expanding MZ provides an increasingly permissive anatomical substrate for the elaboration of nerve‐associated profiles. In this context, increasing cortical S100‐positive area likely reflects integration of neural elements into a progressively organised cortical architecture (Faraj et al., 2019; Honeycutt et al., 2023; Tarnick et al., 2023; Yamaguchi et al., 2025).

### 4.2 Perivascular dominance supports neurovascular co‐development

Intrarenal nerve‐associated profiles were consistently identified in close association with developing renal vessels, suggesting that neural expansion within the cortex follows the spatial organisation of the vascular tree. This neural network expands with advancing gestation towards the outer cortex and renal capsule, positioning neural elements strategically to influence vascular tone and renin‐related regulatory mechanisms during late foetal maturation (DiBona, 2000; Tiniakos et al., 2004; Torretti, 1982). Complementing these human observations, high‐resolution developmental mapping studies in animal models demonstrate that renal axons closely track renal arteries and arterioles as they extend into the cortex, subsequently branching towards glomerular and tubular targets. These findings reinforce the concept that the vascular system provides both a structural scaffold and spatial template for intrarenal neural wiring (N'Guetta et al., 2024). Taken together, these data support a coherent developmental sequence in which increasing vascularisation promotes perivascular nerve development, resulting in a predominance of perivascular distribution and a measurable increase in cortical nerve‐associated profiles with advancing renal maturity.

### 4.3 Early juxtaglomerular and intraglomerular profiles

The presence of juxtaglomerular and intraglomerular S100‐positive profiles from early gestation should be interpreted cautiously, given the structural complexity of the glomerular vascular pole. This region represents a dense microanatomical interface comprising afferent and efferent arterioles, extraglomerular mesangium and renin‐associated cell populations, where closely apposed structures may appear intraglomerular in thin histological sections (Goligorsky, 2022). One possible explanation is a sectioning or projection effect, whereby nerve‐associated S100‐positive profiles adjacent to the vascular pole appear intraglomerular due to tangential sectioning or clustering of perivascular elements (Li et al., 2021; Pullman, 2019). A second possibility is that S100 immunoreactivity reflects non‐neuronal pole‐associated cell populations, as S100 expression is not entirely restricted to neural tissue and has been reported in juxtaglomerular‐related cells in some species (de Girolamo et al., 2000). Finally, a subset of S100‐positive structures at the glomerular pole may correspond to peripolar cells, which have been described in human kidneys as a distinct cell population closely associated with the juxtaglomerular apparatus. Although their functional role remains incompletely understood, their anatomical localisation and secretory phenotype suggest potential involvement in local glomerular or vascular regulation (Gardiner et al., 1986, 1991; Gardiner & Lindop, 1985).

### 4.4 Future directions

Future studies should combine quantitative morphometric analysis with more specific neural and juxtaglomerular markers to better resolve the cellular identity of S100‐positive structures in the developing human kidney. The use of complementary neural markers and higher resolution or three‐dimensional approaches would help distinguish true intrarenal nerve fibres from non‐neuronal pole‐associated cells. Linking these structural findings to functional readouts relevant to renal and cardiovascular regulation will be essential to clarify the physiological significance of intrarenal innervation during late foetal maturation.

### 4.5 Limitations

Several limitations of this study should be acknowledged. First, the sample size was relatively small, reflecting the limited availability of well‐preserved human foetal kidney tissue across defined gestational windows. However, this constraint is inherent to human developmental studies and was partly mitigated by quantitative, compartment‐resolved analyses and consistent findings across specimens. Second, although S100 is commonly used to visualise Schwann cell‐containing peripheral nerve profiles, it is not neuron‐ or axon‐specific and may be expressed by other cell populations. Consequently, the S100‐positive area measured in this study should not be interpreted as a direct measure of axonal or nerve density. This limitation is particularly relevant to the juxtaglomerular and glomerular vascular pole regions, where several closely apposed neural and non‐neural cell populations may contribute to the observed immunoreactivity. The absence of complementary neuronal or sympathetic markers, such as PGP9.5, neurofilament proteins, neuron‐specific enolase, or tyrosine hydroxylase, precludes definitive cellular and phenotypic characterisation of the S100‐positive profiles. Accordingly, our findings represent a quantitative morphological assessment of S100‐positive nerve‐associated profiles, and future studies using complementary markers and colocalisation approaches are required to confirm their neural identity and functional phenotype. Lastly, the study focused exclusively on cortical compartments, precluding conclusions regarding medullary or hilar innervation patterns.

## 5 CONCLUSIONS

In conclusion, this study provides the first quantitative, compartment‐resolved characterisation of intrarenal S100‐positive nerve‐associated profiles in the human foetal kidney across mid‐ to late gestation. We demonstrate that the expansion of cortical S100‐positive profiles parallels histomorphological cortical maturation and increasing gestational age, with a predominant perivascular distribution that supports a neurovascular co‐development model. Early juxtaglomerular and intraglomerular S100 positivity should be interpreted cautiously, as the glomerular vascular pole is a structurally complex microanatomical hub and a known site where peripolar cells may occur. Although functional implications cannot be directly inferred, these findings establish a quantitative morphological framework for future studies investigating the role of renal innervation in foetal kidney maturation and its potential relevance for postnatal renal and cardiovascular regulation.

## AUTHOR CONTRIBUTIONS

IP and BA‐V conceived and designed the study, performed the histological and morphometric analyses, conducted the statistical analyses, prepared the figures and drafted the manuscript, contributed to study design, assisted with methodological development and data interpretation, and critically revised the manuscript. MB, SS and IM selected and identified the foetal kidney specimens and contributed to data interpretation. MŠ and ST‐D assisted with histological processing and data acquisition. BA‐V supervised the study, contributed to data interpretation and critically revised the manuscript. All authors read and approved the final manuscript.

## FUNDING INFORMATION

This research was supported by the Open Publishing Agreement of the Medical University of Vienna.

## CONFLICT OF INTEREST STATEMENT

The authors declare no conflicts of interest.

## ETHICS STATEMENT

The study protocol is reviewed and approved by the Ethics Committee of the University Clinical Centre of Vojvodina, Novi Sad, Serbia (No. 00‐1160).

## Data Availability

The original contributions presented in the study are included in the article; further inquiries can be directed to the corresponding author.

## References

[joa70260-bib-0001] Andrejić, V.B. , Petrović, I. , Balenović, A. , Milosavljević, I. , Petković, J. , Trivunić, D.S. et al. (2024) Analysis of fetal renal cortex development: cortical maturation index as a new potential guide in fetal renal cortex assessment. Vojnosanitetski Pregled, 81(8), 491–497. Available from: 10.2298/VSP240328047A

[joa70260-bib-0002] Barajas, L. & Wang, P. (1975) Demonstration of acetylcholinesterase in the adrenergic nerves of the renal glomerular arterioles. Journal of Ultrastructure Research, 53(2), 244–253. Available from: 10.1016/S0022-5320(75)80141-9 1195453

[joa70260-bib-0003] de Girolamo, P. , Arcamone, N. & Gargiulo, G. (2000) S100‐like protein in the goldfish (*Carassius auratus*) kidney. An immunohistochemical study. Cell and Tissue Research, 302(1), 135–138. Available from: 10.1007/s004410000250 11079723

[joa70260-bib-0004] DiBona, G.F. (2000) Neural control of the kidney: functionally specific renal sympathetic nerve fibers. American Journal of Physiology. Regulatory, Integrative and Comparative Physiology, 279(5), R1517–R1524. Available from: 10.1152/ajpregu.2000.279.5.R1517 11049831

[joa70260-bib-0005] DiBona, G.F. (2003) Neural control of the kidney. Hypertension, 41(3), 621–624. Available from: 10.1161/01.HYP.0000047205.52509.8A 12623969

[joa70260-bib-0006] Dourou, V. , Lyroudia, K. , Karayannopoulou, G. , Papadimitriou, C. & Molyvdas, I. (2006) Comparative evaluation of neural tissue antigens – neurofilament protein (NF), peripherin (PRP), S100B protein (S100B), neuron‐specific enolase (NSE) and chromogranin‐A (CgA) – in both normal and inflamed human mature dental pulp. Acta Histochemica, 108(5), 343–350. Available from: 10.1016/j.acthis.2006.06.001 16919707

[joa70260-bib-0007] Faraj, R. , Irizarry‐Alfonzo, A. & Puri, P. (2019) Molecular characterization of nephron progenitors and their early epithelial derivative structures in the nephrogenic zone of the canine fetal kidney. European Journal of Histochemistry, 63(3), 3049. Available from: 10.4081/ejh.2019.3049 PMC6763752 31544449

[joa70260-bib-0008] Gardiner, D.S. , Downie, I. , Gibson, I.W. , More, I.A. & Lindop, G.B. (1991) The glomerular peripolar cell: a review. Histology and Histopathology, 6(4), 567–573. 1804435

[joa70260-bib-0009] Gardiner, D.S. & Lindop, G.B. (1985) The granular peripolar cell of the human glomerulus: a new component of the juxtaglomerular apparatus? Histopathology, 9(7), 675–685. Available from: 10.1111/j.1365-2559.1985.tb02853.x 3899894

[joa70260-bib-0010] Gardiner, D.S. , More, I.A. & Lindop, G.B. (1986) The granular peripolar cell of the human glomerulus: an ultrastructural study. Journal of Anatomy, 146, 31–43. PMC1166522 3693060

[joa70260-bib-0011] Gattone, V.H.I. , Evan, A.P. , Overhage, J.M. & Severs, W.B. (1990) Developing renal innervation in the spontaneously hypertensive rat: evidence for a role of the sympathetic nervous system in renal damage. Journal of Hypertension, 8(5), 423–428. 10.1097/00004872-199005000-00005 2163416

[joa70260-bib-0012] Goligorsky, M.S. (2022) Emerging insights into glomerular vascular pole and microcirculation. Journal of the American Society of Nephrology, 33(9), 1641–1648. Available from: 10.1681/ASN.2022030354 PMC9529196 35853715

[joa70260-bib-0013] Honeycutt, S.E. , N'Guetta, P.E.Y. , Hardesty, D.M. , Xiong, Y. , Cooper, S.L. , Stevenson, M.J. et al. (2023) Netrin 1 directs vascular patterning and maturity in the developing kidney. Development, 150(22), dev201886. Available from: 10.1242/dev.201886 PMC10690109 37818607

[joa70260-bib-0014] Jackson, E.K. (2004) Autonomic control of the kidney. In: Robertson, D. , Biaggioni, I. , Burnstock, G. & Low, P.A. (Eds.) Primer on the autonomic nervous system [Internet], 2nd edition. San Diego, CA: Academic Press, pp. 157–161. Available from: 10.1016/B978-012589762-4/50041-4

[joa70260-bib-0015] Johns, E.J. (2013) Autonomic regulation of kidney function. In: Handbook of clinical neurology [Internet]. Amsterdam: Elsevier, pp. 203–214. Available from: 10.1016/B978-0-444-53491-0.00017-1 24095127

[joa70260-bib-0016] Johns, E.J. , Kopp, U.C. & DiBona, G.F. (2011) Neural control of renal function. Comprehensive Physiology, 1(2), 731–767. Available from: 10.1002/j.2040-4603.2011.tb00345.x 23737201

[joa70260-bib-0017] Li, X. , Davis, R.C. , Xu, Y. , Wang, Z. , Souma, N. , Sotolongo, G. et al. (2021) Deep learning segmentation of glomeruli on kidney donor frozen sections. Journal of Medical Imaging (Bellingham, Wash.), 8(6), 067501. Available from: 10.1117/1.JMI.8.6.067501 PMC8685284 34950750

[joa70260-bib-0018] N'Guetta, P.E.Y. , McLarnon, S.R. , Tassou, A. , Geron, M. , Shirvan, S. , Hill, R.Z. et al. (2024) Comprehensive mapping of sensory and sympathetic innervation of the developing kidney. Cell Reports, 43(10), 114860. Available from: 10.1016/j.celrep.2024.114860 PMC11616766 39412983

[joa70260-bib-0019] Page, W.V. , Perlman, S. , Smith, F.G. , Segar, J.L. & Robillard, J.E. (1992) Renal nerves modulate kidney renin gene expression during the transition from fetal to newborn life. American Journal of Physiology. Regulatory, Integrative and Comparative Physiology, 262(3), R459–R463. Available from: 10.1152/ajpregu.1992.262.3.R459 1373041

[joa70260-bib-0020] Pokarna, D.J. , Kshitija, K. & Saritha, S. (2019) Histogenesis of human fetal kidney from 14 weeks to 36 weeks: a study. International Journal of Research in Medical Sciences, 7(11), 4330–4334. Available from: 10.18203/2320-6012.ijrms20195011

[joa70260-bib-0021] Pullman, J.M. (2019) New views of the glomerulus: advanced microscopy for advanced diagnosis. Frontiers in Medicine, 6, 37. Available from: 10.3389/fmed.2019.00037 PMC6416220 30899761

[joa70260-bib-0022] Robillard, J.E. , Guillery, E.N. , Segar, J.L. , Merrill, D.C. & Jose, P.A. (1993) Influence of renal nerves on renal function during development. Pediatric Nephrology, 7(5), 667–671. Available from: 10.1007/BF00852576 8251342

[joa70260-bib-0023] Robillard, J.E. & Nakamura, K.T. (1988) Neurohormonal regulation of renal function during development. American Journal of Physiology. Renal Physiology, 254(6), F771–F779. Available from: 10.1152/ajprenal.1988.254.6.F771 3289401

[joa70260-bib-0024] Ryan, D. , Sutherland, M.R. , Flores, T.J. , Kent, A.L. , Dahlstrom, J.E. , Puelles, V.G. et al. (2018) Development of the human fetal kidney from mid to late gestation in male and female infants. eBioMedicine, 27, 275–283. Available from: 10.1016/j.ebiom.2017.12.016 PMC5828465 29329932

[joa70260-bib-0025] S100 [Internet] . (2026) Available from: https://www.pathologyoutlines.com/topic/stainss100.html

[joa70260-bib-0026] Sariola, H. , Saarma, M. , Sainio, K. , Arumäe, U. , Palgi, J. , Vaahtokari, A. et al. (1991) Dependence of kidney morphogenesis on the expression of nerve growth factor receptor. Science, 254, 571–573. Available from: 10.1126/science.1658930 1658930

[joa70260-bib-0027] Schneider, C.A. , Rasband, W.S. & Eliceiri, K.W. (2012) NIH image to ImageJ: 25 years of image analysis. Nature Methods, 9(7), 671–675. Available from: 10.1038/nmeth.2089 PMC5554542 22930834

[joa70260-bib-0028] Slotkin, T.A. , Levant, B. , Orband‐Miller, L. , Queen, K.L. & Stasheff, S. (1988) Do sympathetic neurons coordinate cellular development in the heart and kidney? Effects of neonatal central and peripheral catecholaminergic lesions on cardiac and renal nucleic acids and proteins. The Journal of Pharmacology and Experimental Therapeutics, 244(1), 166–172. Available from: 10.1016/S0022-3565(25)24228-8 2447274

[joa70260-bib-0029] Slotkin, T.A. , Whitmore, W.L. , Orband‐Miller, L. , Queen, K.L. & Haim, K. (1987) Beta adrenergic control of macromolecule synthesis in neonatal rat heart, kidney and lung: relationship to sympathetic neuronal development. The Journal of Pharmacology and Experimental Therapeutics, 243(1), 101–109. Available from: 10.1016/S0022-3565(25)39205-0 2444697

[joa70260-bib-0030] Smith, F.G. , Klinkefus, J.M. & Robillard, J.E. (1992) Effects of volume expansion on renal sympathetic nerve activity and cardiovascular and renal function in lambs. American Journal of Physiology. Regulatory, Integrative and Comparative Physiology, 262(4), R651–R658. Available from: 10.1152/ajpregu.1992.262.4.R651 1566931

[joa70260-bib-0031] Smith, F.G. , Smith, B.A. , Guillery, E.N. & Robillard, J.E. (1991) Role of renal sympathetic nerves in lambs during the transition from fetal to newborn life. The Journal of Clinical Investigation, 88(6), 1988–1994. Available from: 10.1172/JCI115525 PMC295784 1836470

[joa70260-bib-0032] Steiner, J. , Bernstein, H.G. , Bielau, H. , Berndt, A. , Brisch, R. , Mawrin, C. et al. (2007) Evidence for a wide extra‐astrocytic distribution of S100B in human brain. BMC Neuroscience, 8(1), 2. Available from: 10.1186/1471-2202-8-2 PMC1769505 17199889

[joa70260-bib-0033] Tarnick, J. , Elhendawi, M. , Holland, I. , Chang, Z. & Davies, J.A. (2023) Innervation of the developing kidney in vivo and in vitro. Biology Open, 12(8), bio060001. Available from: 10.1242/bio.060001 PMC10411870 37439314

[joa70260-bib-0034] Tiniakos, D. , Anagnostou, V. , Stavrakis, S. , Karandrea, D. , Agapitos, E. & Kittas, C. (2004) Ontogeny of intrinsic innervation in the human kidney. Anatomy and Embryology, 209(1), 41–47. Available from: 10.1007/s00429-004-0420-3 15480775

[joa70260-bib-0035] Torretti, J. (1982) Sympathetic control of renin release. Annual Review of Pharmacology and Toxicology, 22, 167–192. Available from: 10.1146/annurev.pa.22.040182.001123 6282185

[joa70260-bib-0036] Yamaguchi, M. , Yamaguchi, H. , Smith, J.P. , de Almeida, L.F. , Matsuoka, D. , Martini, A.G. et al. (2025) Renin cells drive kidney neurovascular development and arterial remodeling when renin activity is deficient. *bioRxiv*. 2025.07.08.663706 10.1101/2025.07.08.663706

